# Selective inhibition of apicoplast tryptophanyl-tRNA synthetase causes delayed death in *Plasmodium falciparum*

**DOI:** 10.1038/srep27531

**Published:** 2016-06-09

**Authors:** Charisse Flerida A. Pasaje, Vanessa Cheung, Kit Kennedy, Erin E. Lim, Jonathan B. Baell, Michael D. W. Griffin, Stuart A. Ralph

**Affiliations:** 1 Department of Biochemistry and Molecular Biology, Bio21 Molecular Science and Biotechnology Institute, The University of Melbourne, Victoria 3010, Australia; 2 Medicinal Chemistry, Monash Institute of Pharmaceutical Sciences, Monash University, 399 Royal Parade, Parkville, 3052 Victoria, Australia

## Abstract

The malaria parasite *Plasmodium falciparum* relies on efficient protein translation. An essential component of translation is the tryptophanyl-tRNA synthetase (TrpRS) that charges tRNA^trp^. Here we characterise two isoforms of TrpRS in *Plasmodium;* one eukaryotic type localises to the cytosol and a bacterial type localises to the remnant plastid (apicoplast). We show that the apicoplast TrpRS aminoacylates bacterial tRNA^trp^ while the cytosolic TrpRS charges eukaryotic tRNA^trp^. An inhibitor of bacterial TrpRSs, indolmycin, specifically inhibits aminoacylation by the apicoplast TrpRS *in vitro*, and inhibits *ex vivo Plasmodium* parasite growth, killing parasites with a delayed death effect characteristic of apicoplast inhibitors. Indolmycin treatment ablates apicoplast inheritance and is rescuable by addition of the apicoplast metabolite isopentenyl pyrophosphate (IPP). These data establish that inhibition of an apicoplast housekeeping enzyme leads to loss of the apicoplast and this is sufficient for delayed death. Apicoplast TrpRS is essential for protein translation and is a promising, specific antimalarial target.

*Plasmodium falciparum*, the causative agent of the most severe form of malaria in humans, undergoes an intraerythrocytic replication process that results in the clinical manifestations of the disease. Because rapid, constitutive growth and expansion is central to the intraerythrocytic growth stages, the parasite is highly dependent on continued protein synthesis. In addition to protein synthesis in the cytosol, *Plasmodium* parasites depend on two additional organellar translation machineries found in the mitochondrion as well as in a relic plastid known as the apicoplast. These endosymbiotic organelles have bacterial translation machineries that reflect their prokaryotic origins and are sensitive to well-characterised inhibitors of bacterial translation, including the antibiotics doxycycline^1^,^2^,^3^, clindamycin^4^, and azithromycin^5^. While inhibitors of cytosolic protein biosynthesis are fast acting^6^, antibiotics targeting apicoplast translation bring about parasite death only in the replicative cycle subsequent to treatment, a phenomenon known as “delayed death”^7^,^8^,^9^. The mechanism of delayed death is unknown but seems to result from the parasite initially maintaining metabolic pathways despite damage to housekeeping pathways, followed by ablation of the apicoplast and its metabolism in the second cycle^3^.

The recurring emergence and spread of resistance against current and previous antimalarial drugs means that identification of inhibitors with novel modes of action remains a priority. While several translation inhibitors that target the parasite ribosome are clinically used antimalarials^1^,^2^, there has been recent interest in compounds that block protein synthesis through inhibition of aminoacyl-tRNA synthetases (aaRS)^10^,^11^,^12^,^13^,^14^,^15^,^16^,^17^. aaRSs ensure the accuracy of protein biosynthesis by attaching amino acids to their cognate tRNA molecule and editing mischarged tRNAs. Nuclear-encoded *P. falciparum* aaRSs fulfil the requirements for protein synthesis in the cytosol, the mitochondrion, and the apicoplast^15^,^18^. Since aaRSs bind and charge substrates with high specificity a separate enzyme is required for each amino acid and for each organelle, with a few exceptions. In several cases the parasite makes up for a shortfall in organelle-specific aaRS enzymes by targeting a single enzyme to multiple subcellular destinations^15^,^19^. Although translation in the mitochondrion is not well understood, it is thought that aminoacylated tRNAs are imported into the organelle as observed in some other protist parasites^20^,^21^,^22^.

The tryptophanyl-tRNA synthetase (TrpRS) is a class I aaRS characterised by a Rossman-fold catalytic domain containing canonical HIGH and KMSKS motifs^23^,^24^. The *P. falciparum* nuclear genome contains two putative *TrpRS* genes—we show here that one encodes an apicoplast targeted *TrpRS* with preferential activity for charging bacterial tRNA while the other transcribes a cytosolic *TrpRS* with preference for eukaryotic tRNA. Recently, the ligand-free and ligand-bound crystal structures of this latter, cytosolic TrpRS were solved^25^,^26^. Despite the high degree of structural similarity to the *Homo sapiens* orthologue, differences in conformational changes upon ligand binding and insertions within the parasite protein were observed which might allow selective inhibition^26^. In the current study, we investigated several putative inhibitors of TrpRS and show that one, indolmycin, specifically inhibits the apicoplast TrpRS and kills parasites in culture. Indolmycin produces a delayed death phenotype characteristic of apicoplast inhibitors, disrupts apicoplast segregation, and its growth inhibition is reversible by complementing apicoplast metabolism through exogenous addition of the apicoplast product isopentenyl pyrophosphate (IPP). These results confirm the apicoplast-specificity of the delayed death phenomenon, and highlight the potential of the apicoplast *Pf*  TrpRS as a potential antimalarial drug target.

## Results

### Nuclear-encoded *P. falciparum* TrpRS isoforms are localised to the cytosol and the apicoplast

**Figure 1:**
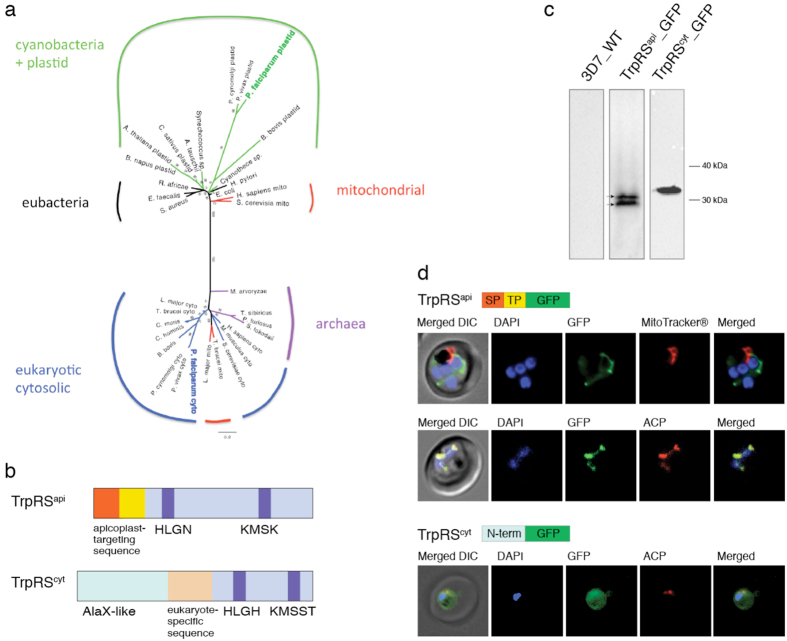
*P. falciparum* encodes cytosolic and apicoplast TrpRS isoforms. (**a**) A Phylogenetic tree inferred from a TrpRS alignment indicates that *Pf*  TrpS^cyt^ is related to eukaryotic TrpRSs, whereas *Pf*  TrpRS^api^ branches with other plastid and bacterial TrpRSs, away from other eukaryotic enzymes. (**b**) Domain organisation of *P. falciparum* TrpRS^api^ and TrpRS^cyt^. The conserved HIGH and KMSKS residues are indicated in purple. *Pf*  TrpRSs bear unique N-terminal sequences with varying functions. In red is the signal peptide portion and yellow is the transit peptide (TP) portion of the apicoplast trafficking sequence. Blue and orange highlight eukaryote-specific sequences. (**c**) Immunoblot of 3D7 *P. falciparum* expressing exogenous TrpRS protein isoforms fused to GFP. Arrows indicate two TrpRS^api^ protein products. (**d**) The first panel shows live cell fluorescence microscopy of TrpRS^api^_GFP and live mitochondrial staining, while the second and third panels show IFAs of *P. falciparum* TrpRS^api^_GFP and TrpRS^cyt^_GFP in relation to the apicoplast marker ACP. Schematic representations of transfected plasmids are indicated above each panel. ACP, acyl-carrier protein; DIC, differential interference contrast.

Results of bioinformatics analyses revealed that the *P. falciparum* nuclear genome encodes two putative *TrpRS* genes. Located on chromosomes 12 and 13, the predicted mature coding sequences are 1680 bp (PlasmoDB ID: PF3D7_1251700) and 1899 bp (PlasmoDB ID: PF3D7_1336900). These genes, which we refer to hereafter as TrpRS^api^ and TrpRS^cyt^ respectively, were used in searches to identify further *TrpRS* genes that were used to construct multiple sequence alignments. Maximum likelihood phylogenetic trees inferred from these alignments revealed that the two *Plasmodium* TrpRSs have very different evolutionary origins (Fig. 1a). While the TrpRS^cyt^ is grouped with other eukaryotic cytosolic TrpRS enzymes, the TrpRS^api^ clusters with bacterial and other plastid TrpRS sequences (Fig. 1a). This bacterial origin is consistent with the presumed endosymbiotic origin of the apicoplast-localised TrpRS, but we cannot confidently determine whether the apicoplast TrpRS^api^ is derived specifically from the original cyanobacterial ancestor of plastids. This alignment did not make for robust phylogenetic reconstruction, and other TrpRS phylogenies have previously been subject of conflicting interpretation. Our phylogenies were unstable based on species selection, tree-building methods, or on the inclusion or exclusion of regions of sequence alignment. Nonetheless, the TrpRS^api^ consistently grouped with other bacterial TrpRSs, while the TrpRS^cyt^ consistently grouped with other eukaryotic, cytosolic TrpRSs, consistent with their assumed evolutionary ancestry.

Apicoplast-targeted products are characterised by an N-terminal trafficking sequence that consist of a signal peptide for trafficking to the ER and a transit peptide for post-translational protein routing to the apicoplast^27^. Manual inspection of the two TrpRS isoforms, as well as prediction using the PlasmoAP and PATS software revealed that TrpRS^api^ bears an approximately 60 amino acid apicoplast trafficking leader sequence (Fig. 1b) which is not conserved with other TrpRSs outside the genus. Although TrpRS^cyt^ also bears a divergent N-terminal sequence, this region has been previously shown to represent an unusual alanyl-tRNA synthetase editing domain (AlaX) followed by a eukaryote-specific extension^25^,^26^ (Fig. 1b).

Subcellular localisation of TrpRS^api^ and TrpRS^cyt^ was verified by fusing the first 60 amino acids of each protein isoform to a C-terminal GFP and expressing these fusion-proteins in *P. falciparum* blood stage parasites. Immunoblot confirmed the expression of the fusion proteins. The TrpRS^cyt^ protein had a motility consistent with the expected mass (33.6 kDa), while the TrpRS^api^ exhibits a doublet band, consistent with the presence of products lacking the signal peptide but before (31.8 kDA) and after (29.4 kDA) processing of the transit peptide (Fig. 1c). Identification of organelle-specific protein localisation was carried out both via live cell fluorescence microscopy and immunofluorescence assay (IFA) using an antibody raised against an apicoplast-targeted protein, ACP^28^. Figure 1d shows colocalisation of GFP with endogenous ACP in the TrpRS^api^ parasite line, validating an apicoplast localisation of the protein. This signal is distinct from the mitochondrial structure (Fig. 1d) that undergoes morphological transformations similar to the apicoplast throughout the parasite life cycle^29^. Analysis of the TrpRS^cyt^-GFP expressing parasite line revealed a cytosolic signal distribution with fluorescence excluded from the nucleus and the digestive vacuole (Fig. 1d), consistent with a previous report of localisation based on TrpRS^cyt^ antiserum^25^. These data indicate that *Plasmodium* parasites possess two TrpRS enyzmes–one bacterial-like TrpRS targeted to the apicoplast and one eukaryotic-like TrpRS targeted to the cytosol.

### Characterisation of TrpRS^api^ and TrpRS^cyt^ by steady state kinetics

To characterise the enzymatic activity of the *P. falciparum* TrpRSs a version of TrpRS^api^_180–1683bp_ with the targeting presequence removed was recombinantly expressed in *E. coli* with a C-terminal hexa-histidine (6x His) tag. A truncated TrpRS^cyt^_687–1896bp_ lacking the *Plasmodium*-specific N-terminal extension but with the eukaryote-specific sequence retained^26^ was also expressed and purified. SDS-PAGE and mass spectrometry results confirm the production of soluble TrpRS^api^ and TrpRS^cyt^ proteins, isolated by Ni-NTA affinity chromatography (Supplementary Fig. 1). TrpRS^api^ and TrpRS^cyt^ aminoacylation activities were determined by measuring the amount of ^3^H- and ^14^C-labelled tryptophan incorporated into the aminoacylated Trp-tRNA^trp^ product. It is known that inorganic pyrophosphate (PPi) generated during the aminoacylation reaction inhibits catalysis by a number of aaRSs^30^. In this study, aminoacylation was carried out in the presence of PPiase to prevent build-up of PPi.

The apparent kinetic parameters of *P. falciparum* TprRS enzymes were investigated for three substrates, ATP, tryptophan, and tRNA, by fitting the initial rate of aminoacylation as a function of substrate concentration to the Michealis-Menten equation (Table 1 and Supplementary Fig. 1). TrpRS^cyt^ charged tryptophan and ATP with an apparent K*m* of 15.5 ± 0.6 and 622 ± 0.3 μM, respectively. We then tested for TrpRS aminoacylation using tRNA from *E. coli, S. cerevisiae*, and *P. falciparum.* Charging of the eukaryotic-type *Pf*  TrpRS^cyt^ was more efficient using yeast tRNA (K_*m*_^app^ = 0.23 ± 0.05 μM) compared to *E. coli* tRNA, but enzyme activity was considerably increased when the assay was carried out using *P. falciparum* tRNA (K_*m*_^app^ = 0.02 ± 0.004 μM).

Consistent with the bacterial origin of the TrpRS^api^, the reverse pattern was seen for this enzyme: it preferentially charged bacterial tRNA (K_*m*_^app^ = 0.12 ± 0.03 μM) over yeast tRNA but aminoacylated its natural substrate, *Plasmodium* tRNA, with 3-fold higher efficiency. Furthermore, the enzyme actively charged tryptophan (K_*m*_^app^ = 5.3 ± 1.2 μM) comparable with bacterial enzymes^31^. We also attempted to determine the K_m_^app^ of TrpRS^api^ for ATP, but inconsistency between biological replicates prevented us from establishing an accurate estimate.

Comparison of the kinetic values between the two enzymes showed a profound difference when charging *P. falciparum* tRNA, with the rate of aminoacylation of TrpRS^cyt^ for *P. falciparum* tRNA observed to be 15-fold greater than that of TrpRS^api^.

These findings show that the two *Pf*  TrpRS enzymes efficiently catalyse the aminoacylation of tryptophan onto its substrates. Furthermore, consistent with the substrate they encounter within the cell, the TrpRS^api^ preferentially charges tRNA from a bacterial source, whereas TrpRS^cyt^ favours tRNA from a eukaryotic source.

### TrpRS^api^ complements a TrpRS mutant *E. coli*

**Figure 2:**
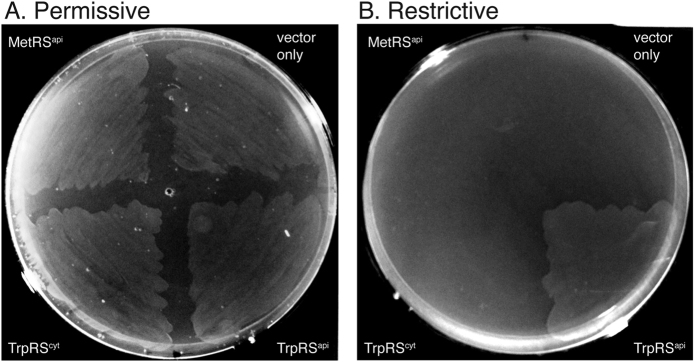
Functional complementation of *E. coli* KY4040. Electrocompetent cells were transformed with MetRS^api^, TrpRS^cyt^_AVA0421, TrpRS^api^_pColdIV, and an empty pColdIV vector. Cells were grown at 37 °C in M9 minimal agar with tryptophan supplementation (0.05 μg/μL) at the permissive condition, and without tryptophan for the restrictive condition.

Functional complementation of an *E. coli TrpRS* mutant was carried out to further validate the activity of recombinant *P. falciparum* TrpRSs. The *E. coli* KY4040 strain harbours an unstable TrpRS that requires high concentrations of L-tryptophan and ATP to function^32^,^33^. KY4040 transformed with an empty expression vector was viable in permissive growth conditions containing 0.05 μg/μL tryptophan (Fig. 2a), but not in repressive conditions lacking tryptophan (Fig. 2b). This defect in KY4040 was not restored by expression of the eukaryotic-type *Pf*  TrpRS^cyt^, but was successfully complemented by expression of *Pf*  TrpRS^api^, which restored bacterial growth in medium lacking tryptophan (Fig. 2b). These findings are in agreement with our kinetic data on aminoacylation, indicating that the *Pf*  TrpRS^api^ but not *Pf*  TrpRS^cyt^ can efficiently recognise and charge bacterial tRNA.

### Bacterial-type TrpRS inhibitors arrest intraerythrocytic *P. falciparum*

TrpRS inhibitors^34^,^35^,^36^,^37^,^38^ were identified via the literature and screened according to their pharmacokinetics properties for inhibition studies in *P. falciparum* (Supplementary Table 1). The tryptophan analogue and natural product, indolmycin, is a well-characterised TrpRS inhibitor and was selected for testing. To find additional compounds with similar structures, analogues of indolmycin were identified *via* compound similarity searches–two additional compounds STK505786 and PH000586 were also selected for testing. A previously characterised bacterial TrpRS inhibitor, referred to as SPECS_C1^38^ was also selected for testing against *P. falciparum* growth (Table 2). Both indolmycin and SPECS_C1 displayed low cytotoxicity to mammalian cells^36^,^37^,^38^. The compounds were tested against *P. falciparum* according to an inhibition assay described previously^39^.

**Figure 3:**
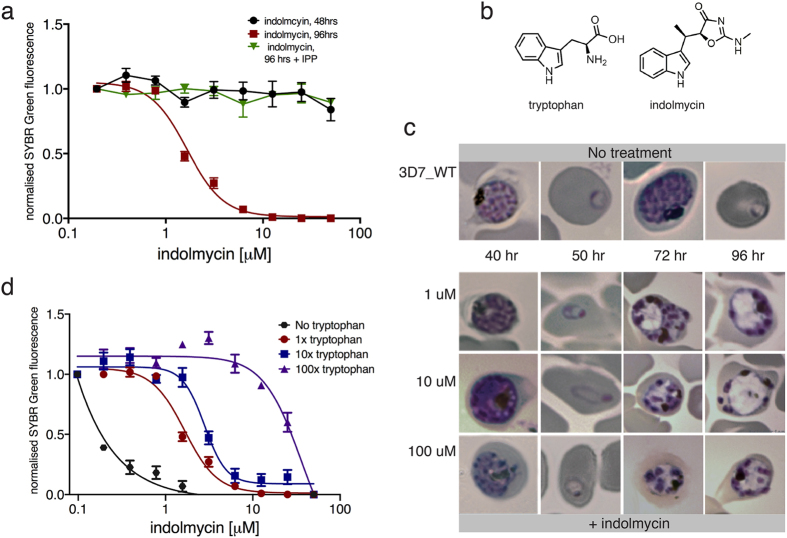
Antiplasmodial activity of indolmycin against *P. falciparum*. (**a**) Dose-response curve from SYBR-Green susceptibility assay determined 48 and 96 hrs after indolmycin treatment with and without IPP supplementation. Indolmycin causes a delayed death effect (inhibition at 96 hrs) that is rescued by IPP. (**b**) Chemical structures of indolmycin and tryptophan. (**c**) Microscopic images of Giemsa-stained parasites with and without indolmycin. (**d**) Dose-response curve from SYBR-Green susceptibility assay determined 96 hrs after indolmycin treatment and with different concentrations of tryptophan. 1x, 10x, and 100x the concentration of the amino acid in complete media corresponds to at 0.02, 0.2, and 2 mM tryptophan, respectively.

Although indolmycin treatment of *P. falciparum* 3D7 and W2*mef* did not result in growth inhibition during the first life cycle, substantial growth arrest was observed at the succeeding replicative cycle (IC_50_ = 1.7 ± 0.5 μM; Fig. 3a–c and Table 2). This inhibition of parasite growth only in the second cycle after treatment is characteristic of apicoplast inhibition^8^. The delayed death phenotype was confirmed from microscopic analysis of Giemsa-stained parasites treated with indolmycin. Even at the highest concentration of indolmycin assayed, parasites were able to progress through the first life cycle exhibited through normal cell division. In the next life cycle, an apparent morphological defect manifested as pale and vacuolated parasite forms were observed (Fig. 3c). These findings suggest that the bacterial-TrpRS inhibitor, indolmycin, kills *P. falciparum* by targeting the apicoplast housekeeping function. In an effort to understand what structural features of indolmycin might be important for biological activity, commercially available analogues were sourced and tested against malaria parasites. It was determined that replacing the oxazolone side chain of indolmycin with an α-hydroxyester resulted in a very large decrease in antimalarial activity (PH000586 IC_50_ ≈ 100 μM at both 48 and 96), whereas α-hydroxycarboxylic acid led to complete loss of inhibition (Summarised in Table 2). Due to the paucity of available, close analogues of indolmycin, finer details on structure-activity relationship requirements for optimum activity would require a synthetic investigation.

One of the analysed compounds, SPECS_C1, killed parasites at a low μM range (IC_50_ = 14.3 ± 3.3 μM Table 2), but resulted in lysis of erythrocytes at similar concentrations, so its specificity for TrpRS in this system is doubtful. SPECS_C1 was identified as a TrpRS inhibitor using *in silico* structure-based virtual screens, and aside from analysis of its effects on bacterial culture turbidity, the compound has not been characterised against a wide range of eukaryotic culture conditions^38^.

### Increasing tryptophan concentration rescues indolmycin-induced inhibition of *P. falciparum*

Given the structural similarity between indolmycin and tryptophan, we hypothesise that the compound interferes with tryptophan processing in the parasites. To test this hypothesis, proliferation of parasites treated with indolmycin and cultured at different concentrations of tryptophan were determined from growth-response curves. While parasite viability was not affected in a tryptophan-depleted media source, the addition of indolmycin severely affected parasite growth, resulting in an 8-fold reduction in IC_50_ (Fig. 3d). However, an increase in tolerance to indolmycin toxicity that is directly proportional to the concentration of tryptophan supplemented in the media was observed. The addition of tryptophan to concentration 10x and 100x greater than that found in normal medium resulted in a 1.7- and a 22-fold increase, respectively, in IC_50_ (Fig. 3d). Negative controls with changed glutamine concentrations in the growth medium had no effect on *P. falciparum* sensitivity to indolmycin. (Fig. S2). These findings are consistent with indolmycin competing with tryptophan incorporation in the parasites.

### Indolmycin-treatment abolishes the parasite apicoplast

The delayed death inhibition described above suggests that indolmycin affects growth of *P. falciparum* by targeting the apicoplast. Recently, it has been shown that apicoplast inhibition can be rescued by supplying parasites with abundant exogenous source of an essential apicoplast metabolite, IPP^3^. In this study, IPP rescue was used as a tool to characterise indolmycin-induced parasite toxicity and to validate the target of the compound.

A SYBR Green assay was first used to assess the viability of indolmycin-treated 3D7 *P. falciparum* following IPP supplementation. Based on nuclear replication, we observe a complete rescue of parasite growth when challenged with indolmycin and cultured in the presence of IPP (Fig. 3a and Table 2) even at concentrations of indolmycin well above those normally required to kill all parasites in the absence of IPP.

**Figure 4:**
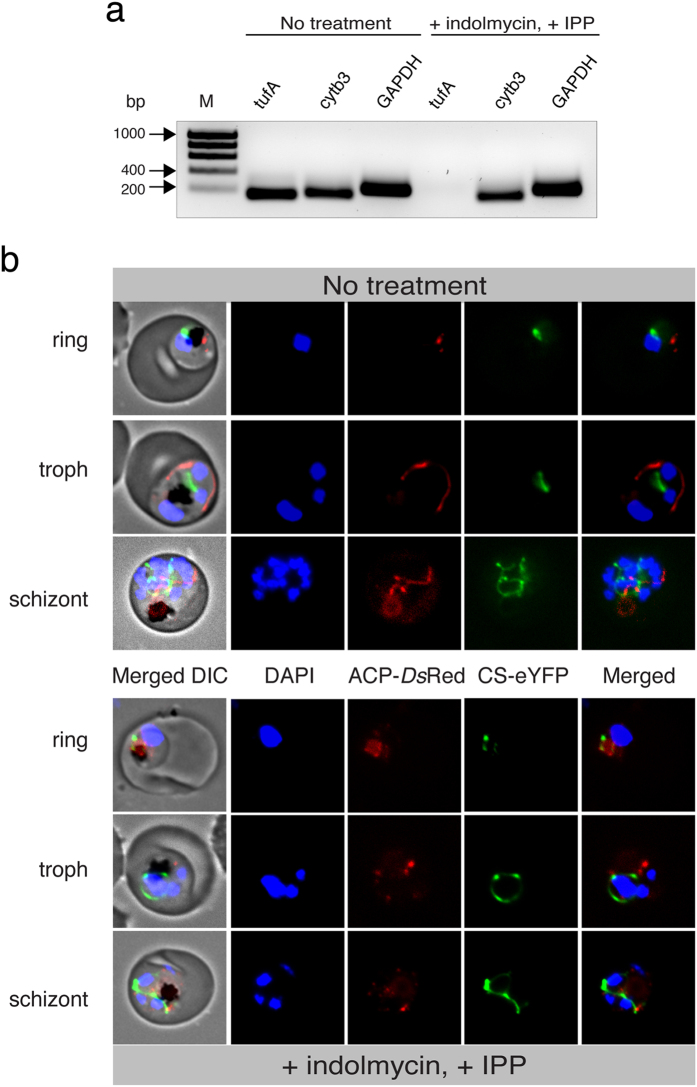
Loss of apicoplast in indolmycin-treated, IPP-rescued parasites. (**a**) Amplification of apicoplast- (*tufA*, 158 bp), mitochondrial- (*cytb3*, 178 bp), and nuclear- (*GAPDH*, 243 bp) encoded genes. Parasites are shown without drug treatment, and five growth cycles after addition of indolmycin and IPP. M = HyperLadder™ 1 kb. (**b**) Live cell fluorescence comparing treated and untreated ACP-*Ds*Red/CS-eYFP *P. falciparum* 3D7. ACP, apicoplast acyl-carrier protein. DIC, differential interference contrast. CS, mitochondrial citrate synthase.

Another indication of apicoplast destruction is the loss of the organellar genome. Genomic analysis was performed to further validate the loss of the apicoplast by indolmycin-treatment. gDNA was extracted at various time course post-drug challenge and genes encoded in the nucleus (*GAPDH*), mitochondria (*cytb3*), and apicoplast (*tufA*) were amplified via PCR. While parasites were able to grow and proliferate normally for at least ten replicative cycles, the apicoplast genome of indolmcyin-challenged parasites was gradually but completely abolished whereas nuclear- and mitochondrial-encoded DNA remained abundant (Fig. 4a).

Apicoplast loss after indolmycin treatment was then visualised via fluorescence microscopy of a transgenic parasite line expressing *Ds*Red and GFP fused to the leader sequences of apicoplast and mitochondrion markers acyl carrier protein (ACP) and citrate synthase (CS), respectively. Parasites treated with both indolmycin (100 μM) and 200 μM IPP proliferated as well as untreated controls, indicating that an apicoplast process is the sole target of indolmycin at these concentrations. Figure 4b shows that, in contrast to untreated samples that exhibit distinctive apicoplast morphology, indolmycin treatment results in apicoplast disruption during the second cycle, with apicoplast proteins dispersing into numerous puncta in the cytoplasm. The mitochondrion maintains its morphological transformation throughout the assay, suggesting that the parasites were viable and replicating normally.

Taken together, these findings show that the parasites were able to bypass the indolmycin-induced toxicity in the absence of a functional apicoplast by using exogenous IPP to maintain isoprenoid synthesis. Together these data indicate that indolmycin affects parasite growth by specifically targeting tryptophan utilisation in the apicoplast.

### Indolmycin inhibition of TrpRS aminoacylation

**Figure 5:**
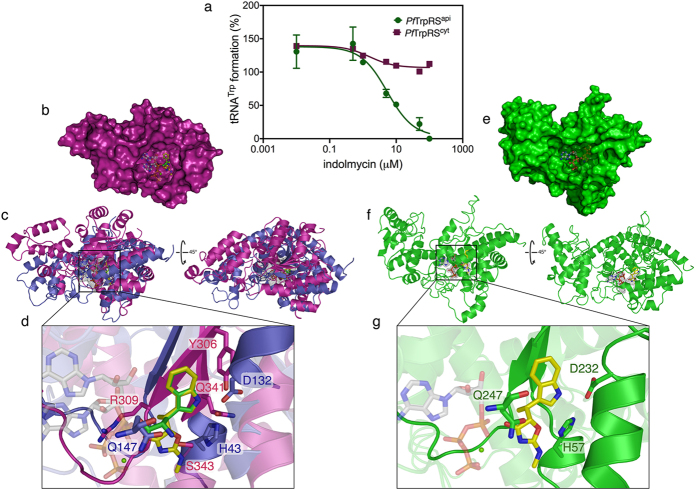
Selective inhibition of *Pf*  TrpRS^api^ but not *Pf*  TrpRS^cyt^ by indolmycin. (**a**) Inhibition of tRNA^trp^ formation by *Pf*  TrpRS^api^ (shown in green) and *Pf*  TrpRS^cyt^ (shown in red) was tested at increasing concentrations of indolmycin. *Pf*  TrpRS^api^ is completely inhibited at higher concentrations, *Pf*  TrpRS^cyt^ is comparatively insensitive. (**b**) Surface representation of chain A of the crystal structure of *P. falciparum* cytosolic TrpRS with bound 5′AMP•tryptophan-indolmycin overlay (PDB: 4J75; shown in magenta) and (**c**) cartoon representations of the protein superimposed with chain A of the crystal structure of *B. stearothermophilus* TrpRS with bound ATP•Mg^2+^•indolmycin (PDB: 5DK4; shown in violet). (**d**) Zoom-in view of ATP•Mg^2+^•indolmycin-tryptophan overlay in the catalytic core of TrpRS. Surface (**e**) and cartoon (**f** ) representations of *P. falciparum* apicoplast TrpRS with bound ATP•Mg^2+^•indolmycin. Note that the iTasser C-score for this model is low (−2.5), indicating global model low quality, although manual inspection indicates a good structural fit in the tryptophan binding region. (**g**) Zoom-in view of ATP•Mg^2+^•indolmycin in the conserved catalytic core of TrpRS. A number of residues within the active site were made transparent to show the ligand and relevant interacting residues. The substrates are shown as sticks. ATP substrates are shown in white, tryptophan is shown in green, and indolmycin is shown in yellow.

The effect of indolmycin on the formation of charged tRNA^trp^ by TrpRS^api^ and TrpRS^cyt^ was determined using the established functional assay for TrpRS activity. Figure 5a reveals that at higher concentrations of indolmycin, tRNA^trp^ formation by TrpRS^api^ was reduced to background levels. Aminoacylation of TrpRS^cyt^ on the other hand was only slightly inhibited. These results are consistent with the *ex vivo* proliferation assays which support an apicoplast target for indolmycin.

The nature of TrpRS inhibition by indolmycin was explored using the recently determined crystal structure of *Pf*  TrpRS^api^ homologue (5DK4; 33% sequence homology) from *Bacillus stearothermophilus* with bound indolmycin, ATP, and magnesium^40^ superimposed with the structure of the cytosolic *Plasmodium* TrpRS (4J75; Fig. 5b–d) with bound charged tryptophan^26^. A structural alignment was performed by overlaying the indole moiety of indolmycin with that of tryptophan. Comparing the tryptophan binding pockets of *Bs*TrpRS and the (indolmycin-insensitive) cytosolic *Pf*  TrpRS in this way reveals significant structural differences. In the case of *Bs*TrpRS, the indole nitrogen of indolmycin forms a hydrogen bond with D132, while in *Pf*  TrpRS the indole nitrogen of the tryptophan derivative is hydrogen bonded to the sidechain hydroxyl groups of Y306 and Q341 (Fig. 5d). These amino acids reside on separate structural elements surrounding the binding site in each enzyme, with (*Bs*)D132 part of the α-helix directly below the binding pocket and (*Pf* )Y306 and Q341 part of the β-sheet adjacent to the binding site. Inspection of the binding mode of indolmycin in this alignment provides some clues as to why *Pf*  TrpRS^cyt^ is not sensitive to inhibition by the compound. In particular, the alignment shows potential steric clashes between the methyl group of indolmycin and the *Pf*  TrpRS^cyt^ protein backbone in the region of the R309, and between the oxazolinone ring and the sidechain of S343 (Fig. 5d).

The program I-TASSER was used to create a three dimensional structure of the apicoplast *Pf*  TrpRS, using the crystal structure of *Bs*TrpRS as a template (Fig. 5e–g). Predicting the *Pf*  TrpRS^api^ structure in this way resulted in a model with an iTasser *C*-score = −2.42 which is lower than the score that correlates with ~90% prediction accuracy for global topology^41^. Nonetheless, the predicted structure shows residues that serve as determinants for indolmycin binding in *Bs*TrpRS to be a similar position in the parasite apicoplast protein–with (*Bs*)H43, D132, and Q147 in equivalent positions to (*Pf *)H57, D232, and Q247, respectively (Fig. 5g). Taken together, these results suggest that whereas the cytosolic *Pf*  TrpRS generates steric clashes that preclude indolmycin binding, the *Pf*  TrpRS^api^ shares the *Bs*TrpRS indolmycin-binding residues, allowing inhibition of tRNA^trp^ aminoacylation and, therefore a block in apicoplast protein translation.

## Discussion

The ongoing emergence and spread of antimalarial drug resistance creates a serious need for the identification of new molecular targets and compounds with distinct antimalarial activities. Inhibitors of protein translation fulfil this requirement, and several tRNA synthetases have been advanced as targets of potential antimalarial compounds^11^,^12^,^13^,^14^,^15^,^16^.

In the current study, we have shown that intraerythrocytic stage malaria parasites have two TrpRS isoforms; TrpRS^cyt^ localises to the cytosol and preferentially aminoacylates eukaryotic tRNA while TrpRS^api^ is targeted to the apicoplast and efficiently aminoacylates bacterial tRNA. Performing aminoacylation reactions with tRNA isolated from *P. falciparum* increased the catalytic efficiency of TrpRS^cyt^ and TrpRS^api^ enzymes. However, because the translation machinery differs between prokaryotic and eukaryotic enzymes, the assay was limited by our inability to specifically isolate apicoplast tRNA. Apicoplast tRNA represents a minor fraction of total purified tRNA and this is likely reflected in the kinetic values obtained for the TrpRS^api^ enzyme. It is known that eukaryotic and prokaryotic tRNAs have different identity elements^42^ that promote substrate-specific binding. This is consistent with the differential activity observed for these substrate using both the apicoplast and cytosolic TrpRSs. The poor cross-recognition between the eukaryotic and bacterial enzymes and substrates explains the persistence of two TrpRS isoforms in *Plasmodium* spp. Though some dual-localised aaRSs have adapted to recognise both organellar and nuclear-encoded tRNAs^15^,^19^ the *Pf*  TrpRS^cyt^ recognises the bacterial type tRNA^trp^ poorly, necessitating retention of compartment-specific TrpRS enzymes in *Plasmodium*.

The retention of a bacterial TrpRS^api^ in *Plasmodium* spp. creates opportunities for parasite-specific inhibition. We explored this susceptibility by testing inhibitors of bacterial TrpRSs against *Plasmodium* growth–indolmycin was the most promising of these. Indolmycin is a natural product and tryptophan analogue isolated from *Streptomyces griseus*^43^,^44^, that affects growth of various gram-positive and -negative bacteria^31^,^37^. One of the tested compounds, SPECS_C1 was discovered from a high-throughput virtual screening of the binding affinity of a compound library against a predicted structural model of *Staphylococcus epidermis* TrpRS, followed by biochemical assays assessing the effect of hit compounds on enzymatic activities^38^. While SPECS_C1 was shown to display dose-dependent inhibition of *S. epidermis* and *S. aureus* TrpRS activity *in vitro*, the observed lack of inhibition against *E. coli* suggests that the effect is species-specific. The apparent lysis of human erythrocytes in our assays at parasite-killing concentrations suggests that further variation to this compound would be necessary before any parasite-specific activity could be investigated.

Previous studies have reported that indolmycin kills bacteria by competitive inhibition of the tryptophan-binding pocket of TrpRS^31^,^37^. Furthermore, two independent studies comparing aminoacylation of the indolmycin-resistant TrpRS to the sensitive gene from *S. coelicolor* and *B. stearothermophilus* showed that indolmycin affects tRNA^trp^ formation as demonstrated by the kinetics of competitive inhibition of tryptophan^31^,^37^. Given the structural similarity between indolmycin and tryptophan, competitive inhibition can also be deduced as the underlying antimalarial mode of action of the compound. This is supported by our data showing that levels of exogenous tryptophan modulate *P. falciparum* sensitivity to indolmycin.

In previous reports exploring the structural basis for the selectivity of indolmycin for bacterial but not eukaryotic TrpRSs, (*Bs*)H43 was implicated in indolmycin sensitivity. Replacement of this amino acid with asparagine resulted in resistance to indolmycin^31^,^35^, potentially by disruption of the hydrogen bond with the oxazolinone ring. Our structural alignment of *Pf*  TrpRS^cyt^ with *Bs*TrpRS shows structural differences surrounding the tryptophan-binding pocket that may underlie the differential binding of indolmycin by each enzyme. The combination of our phylogenetic analysis and homology modelling results suggests that the apicoplast TrpRS may be structurally homologous to bacterial TrpRSs and therefore shares their sensitivity to indolmycin.

Consistent with the relationship of the *Pf*  TrpRS^api^ to bacterial TrpRSs we found that indolmycin specifically inhibits *Pf*  TrpRS^api^ but not *Pf*  TrpRS^cyt^, and specifically ablates apicoplast function. The only essential product of the apicoplast in blood stage *P. falciparum* is the isoprenoid precursor IPP^3^. Complete rescue of parasite growth inhibition by indolmycin using the apicoplast metabolite IPP suggests that apicoplast protein translation is the only important target of indolmycin in these blood stage parasites. The disappearance of the apicoplast genome and the disruption of apicoplast morphology in these indolmycin treated parasites is further proof of the apicoplast target of this compound.

## Materials and Methods

### *P. falciparum* culture

3D7 and W2*mef P. falciparum* were maintained in a continuous culture consisting of human erythrocytes (O^+^, 2% haematocrit) resuspended in RPMI 1640 with 3.6% sodium bicarbonate and 5% Albumax (complete media), and incubated in a gas mixture consisting of 5% CO_2_, 1% O_2_, and 94% N_2_ at 37 °C^45^. Parasitemia was determined every 48 hours through microscopic examination of blood smears fixed in absolute methanol and stained with 10% Giemsa solution.

Transfection of *P. falciparum* was carried out by electroporation as described previously^46^. Briefly, 100 μg of purified plasmid DNA was resuspended in warm TE buffer and cytomix (120 mM KCl, 0.15 mM CaCl_2_, 2 mM EGTA, 5 mM MgCl_2_, 10 mM K_2_HPO_4_/KH_2_PO_4_ pH 7.6, 25 mM HEPES pH 7.6). Synchronous ring-stage parasites (5–10% parasitemia) were added to the plasmid and electroporated at 0.31 kV and 950 μF in a 0.2 cm cuvette. Transfectants were cultured in complete media with 20 nm WR92210, and viable parasites were observed in cultures after three weeks.

To monitor organellar morphology in indolmycin treated parasites, we used a double transfectant parasite line (3D7) expressing the apicoplast acyl-carrier protein (ACP) fused to *Ds*Red and mitochondrial citrate synthase (CS) fused to eYFP^29^. These parasites were a kind gift from Professor Geoffrey McFadden (The University of Melbourne). The deleterious effect of drug treatment was overcome by maintaining the parasites in complete media supplemented with 200 μM IPP following a method described previously^3^.

### Bioinformatic analyses

Full-length sequences of putative TrpRS^api^ (PlasmoDB ID: PF3D7_1251700) and TrpRS^cyt^ (PlasmoDB ID: PF3D7_1336900) were obtained from PlasmoDB^47^. Prediction of an apicoplast-trafficking presequence was performed using PlasmoAP^47^,^48^ and Predict Apicoplast-Targeted Sequences (PATS)^49^. Multiple sequence comparison of the TrpRS protein in *Plasmodium* species and other organisms was carried out using clustalOmega^50^ and were manually adjusted and edited using Jalview^51^. Maxiumum likelihood trees were inferred from this alignment using PhyML^52^ with 1,000 bootstrap replicates performed.

### Cloning of plasmids

N-terminal fragments of TrpRS^api^_1–180bp_ and TrpRS^cyt^_1–180bp_ were synthesised with *Xho*I and *Xma*I restriction sites (BioBasic Inc), and cloned into pGlux for episomal expression in 3D7 *P. falciparum*. This vector contains a *green fluorescence protein* (*GFP*) and the *human dihydrofolate reductase* (*hDHFR*) that confers resistance to WR99210. Plasmid sequences were confirmed by Sanger sequencing (Australian Genome Research Facility Ltd).

A version of TrpRS^api^_180–1683bp_ that lacks the N-terminal trafficking sequence was codon optimised, synthesised with *BamH*I and *Hind*III restriction sites (BioBasic Inc), and cloned into and pColdIV for complementation in *E. coli* KY4040 and pET-21a(+) that allow in-frame fusion of a C-terminal polyhistidine tag for bacterial protein expression. Construct sequences were confirmed by Sanger sequencing. For comparison of enzymatic activity, a glycerol stock of TrpRS^cyt^_687–1896bp_ was obtained from Wim G. Hol at the Seattle Structural Genomics Center for Infectious Disease (SSGCID) and Wes Van Voorhis at the Center for Emerging and Re-emerging Infectious Diseases (CERID).

### Microscopy

Imaging of live cells was performed by staining infected erythrocytes with 20 nM MitoTracker® (Thermo Fisher Scientific) which stains the mitochondrion and 0.5 μg/mL of DAPI to visualise the nucleus. Briefly, 500 μL of parasite culture was pelleted, resuspended in MitoTracker®, and washed twice with PBS (1x), before final staining with DAPI. For visualisation of ACP-*Ds*Red/CS-eYFP 3D7 *P. falciparum*, nuclear staining was carried out prior to fluorescence microscopy.

Immunofluorescence assay (IFA) in solution was carried out to analyse protein subcellular localisation in intraerythrocytic parasites. Infected erythrocytes (8–10% parasitemia) were fixed in PBS (1x) containing 4% (v/v) paraformaldehyde and 0.0075% (v/v) glutaraldehyde for 30 min, permeabilised with 0.1% (v/v) Triton X-100 for 10 min, and blocked with 3% (w/v) BSA for 30 min. Cells were pelleted and incubated for one hr in blocking solution with mouse anti-GFP and rabbit anti-ACP as primary antibodies. Subsequent incubation was carried out in Alexa Fluor® 488 and 594-conjugated anti-mouse and anti-rabbit as secondary antibodies. Cells were washed with 500 mg/mL of DAPI before final resuspension in DABCO and PBS (1x).

Fluorescence microscopy was performed using the Zeiss Axioplan 2 imaging and Leica SP5 Confocal imaging platforms. Images were processed using ImageJ^53^.

### Immunoblotting

To verify the expression of TrpRS^api^_pGlux and TrpRS^cyt^_pGlux in *P. falciparum*, erythrocytes infected with trophozoite stage parasites (8–10% parasitemia) were lysed with 0.01% saponin and complete™, EDTA-free Protease Inhibitor (Roche) resuspended in PBS (1x) for 10 minutes at 4 °C. Cells were pelleted and resuspended in PBS (1x) and sample loading buffer (SLB; 3x) containing sodium dodecyl sulfate (SDS) and dithiothreitol (DTT).

Samples were loaded onto Mini-PROTEAN^®^ TGX™ Precast Gels (Bio-Rad) in standard Tris-glycine buffer, and polyacrylamide gel electrophoresis separated proteins were transferred to polyvinylidene fluoride (PVDF) membranes and blocked with 10% (w/v) skim milk in TBS/Tween. Membrane-bound proteins were incubated in mouse anti-GFP and rabbit anti-mouse HRP-conjugated primary and secondary antibodies, respectively. Following a 5-minute incubation in SuperSignal® West Pico Chemiluminescent (Thermo Fisher Scientific) substrate, protein blots were analysed using the Gel Pro Analyzer 4.0.

### Protein expression and purification

*E. coli* Rosetta that contains the pRARE plasmid was transformed with 5 ng of TrpRS^api^_pET-21a(+) and TrpRS^cyt^_AVA0421 plasmids and plated on LB agar plates supplemented with ampicillin and chloramphenicol. A single colony was expanded in 0.5 L of ZYP-5052 auto-induction media^54^ and incubated at 37 °C for 18 hours with proper aeration. Isopropyl β-D-1-thiogalactopyranoside (IPTG; 1 mM) was added to TrpRS^api^_pET-21a(+) and the culture was incubated for two more hours.

Cells were harvested by centrifugation at 6,000 × *g* for 30 mins. Pelleted cells were resuspended in BugBuster® Master Mix (Merck Millipore) with complete™, EDTA-free Protease Inhibitor (Roche) and passed through a French press for complete lysis of cell membrane. TrpRS proteins were purified by nickel-NTA chromatography using an imidazole gradient. Fractions were concentrated and dialysed against 100 mM HEPES using Amicon® Ultra Centrifugal Filters (Merck Millipore) and stored in 10% glycerol at 80 °C. Fractions collected from batch purification and concentrated protein were analysed via SDS-PAGE.

### Protein mass spectrometry

In-gel trypsin digestion was carried out to validate protein expression by mass spectrometry. Purified TrpRS^api^ and TrpRS^cyt^ separated from a protein mixture by SDS-PAGE was gel-excised, destained overnight with 50 mM triethylammonium bicarbonate (TEAB) in acetonitrile, and incubated in 10 mM tris(2-carboxyethyl)phosphine (TCEP) to reduce disulfide bonds and iodoacetamide to alkylate free cysteines. Proteins were digested with proteomic-grade trypsin (Sigma-Aldrich) and peptides were analysed on the Thermo Scientific Orbitrap Elite™ mass spectrometer. Data analysis was carried out using MASCOT v2.4^55^ using a *Plasmodium* subset of UNIPROT as the database with a mass tolerance of 20 ppm and 0.6 Da, 3 possible missed cleavage events, and one variable modification allowing for oxidized Methionine.

### *In vitro* assay for *Pf*  TrpRS activity

Total RNA was extracted from *P. falciparum* 3D7 wild type parasites by TRIzol (Sigma-Aldrich) treatment and tRNA-containing small RNA species were isolated using the PureLink™ miRNA Isolation Kit (Thermo Fisher Scientific).

Aminoacylation of TrpRS^api^ and TrpRS^cyt^ were carried out in 100 mM HEPES, 20 mM MgCl_2_, 30 mM KCl, 2 mM DTT, 0.1 mg/mL BSA, 2U/mL inorganic pyrophosphatase (PPiase, New England BioLabs, ref M0361L), 50 μM cold L-tryptophan (Sigma), 38 μM ^14^C-tryptophan (53.8 mCi/mmol, Perkin Elmer)^1^,^2^ or ^3^H-tryptophan (20.1 mCi/mmol, Perkin Elmer), and varying concentrations of ATP and tRNA from *P. falciparum*, *E. coli* (Sigma-Aldrich, ref R1753) and *Saccharomyces cerevisiae* (Sigma-Aldrich, ref R5636). All assays were carried out at 37 °C and measurements were taken at various timepoints for each protein. Radioactive tRNA^trp^ product was precipitated in trichloroacetic acid (TCA, Chem Supply, ref TA030), captured on Whatman® filter papers presoaked in TCA with 5 mM L-tryptophan, and quantified using a Tri-Carb 4810TR Liquid Scintillation Counter. Kinetic parameters were determined from the Michaelis-Menten and Lineweaver Burk equations.

To determine the kinetics of *Pf*  TrpRS inhibition, an intermediate concentration of tRNA substrate was used to create progress curves in the presence of varying concentrations of the inhibitors (0.1–100 μM indolmycin) following a method described previously^37^. Purified proteins were incubated with the inhibitor for 1 hr at 37 °C and aminoacylation was carried out as described above.

### Complementation of TrpRS mutant *E. coli*

Electrocompetent *TrpRS* mutant *E. coli*, KY4040^32^,^33^ was transformed with 5 ng of TrpRS^api^_181–1683__pColdIV, TrpRS^cyt^_AVA04521, *P. falciparum* apicoplast methionyl-tRNA synthetase (MetRS^api^_pColdIV, PF3D7_1005000), and empty pColdIV vector. Cells were plated on M9 minimal agar plates with (permissive) and without (repressive) tryptophan supplementation (0.05 μg/μL) and incubated at 37 °C.

### Drug susceptibility assays

Inhibitors of TrpRS in other organisms were taken from the literature^34^,^35^,^36^,^37^,^38^,^44^, and substructure search of one of the compounds was performed using a chemical search engine, eMolecules (http://www.emolecules.com/). Four compounds, indolmycin (BioAustralis, ref BIA-I1040), SPECS_C1 (Specs Netherlands, 2-{5-[(6-(ethoxycarbonyl)-5-(4-fluorophenyl)-3-oxo-7-phenyl-5H-[1,3]thiazolo[3,2-a]pyrimidin-2(3H)-ylidene)methyl]-2-furyl}benzoic acid), PH000586 (Vitas M Labs), and STK505786 (Sigma-Aldrich, 2-hydroxy-3-(1H-indol-3-yl) butanoic acid), were received as dry powders (>99% purity), dissolved in DMSO, and stored at either −20 °C or room temperature. In Falcon® 96-Well Flat-Bottom plates, synchronous ring-stage 3D7 and W2*mef P. falciparum* were set up in triplicates and treated with varying concentrations of each compound. Media was replaced every cycle, and growth inhibition was analysed from 0–48 and 0–96 hrs using SYBR Green assay as described previously^56^. Corrected SYBR green fluorescence values were visualised on a scatter plot and IC_50_ values were obtained from the dose-response curves.

Assessment of parasite viability following indolmycin-treatment and IPP supplementation was carried out by performing a proliferation assay with the metabolite added in the media in the second replicative cycle (48–96 hours) of the parasites.

### PCR

Trophozoite-stage parasites from 200 μL of indolmycin- and IPP-treated culture were saponin-lysed, boiled, and freeze-thawed to extract genomic DNA. PCR was performed using MangoTaq™ DNA polymerase and primers that amplify genes in various parasite organelles: *GAPDH* (nuclear) 5′-ATCAAAGGGTGGTAAGGACTGG-3′/5′-AGTGGACCTTCAGCAGCTTTTT-3′, *tufA* (apicoplast) 5′-GATATTGATTCAGCTCCAGAAGAAA-3′/5′-ATATCCATTTGTGTGGCTCCTATAA-3′, *cytb3* (mitochondria) 5′-AGATACATGCACGCAACAGG-3′/5′-TCATTTGACCCCATGGTAAGA-3′.

### Tryptophan rescue assays

Synchronous early ring-stage *P. falciparum* 3D7 parasites were adapted to RPMI 1640 devoid of tryptophan. Tryptophan was added at 0.02, 0.2, and 2 mM corresponding to 1x, 10x, and 100x the concentration of the amino acid in complete media, respectively.

Proliferation rate of tryptophan-starved and -fed parasites treated with different concentrations of indolmycin was assessed using the SYBR Green assay described above. Growth of glutamine-starved and -fed parasites were analysed as controls.

### Structural analysis

Chain A of the crystal structure of *Pf*  TrpRS^cyt^ bound with tryptophan•5′AMP (PDB accession code 4J75) was superimposed with the *B. stearothermophilus* TrpRS with bound indolmcyin•Mg^2+^•ATP (PDB accession code 5DK4) that shares 33% sequence homology with *Pf*  TrpRS^api^. Structural alignment was carried out by manually overlaying the indole moiety of indolmycin (5DK4) with that of tryptophan (4J75). All Figures displaying protein structures were rendered with PyMol software from Delano Scientific (The PyMOL Molecular Graphics System, Version 1.5.0.4 Schrödinger, LLC)^57^.

*Pf*  TrpRS^api^_61–559aa_ without the presequence region was modeled by I-TASSER^41^,^58^ using PDB 5DK4 as a preferred template without sequence alignment. The predicted protein structure (*C*-score = −2.42) in complex with ATP•Mg^2+^•indolmycin from PDB 5DK4 was then visualised with PyMol.

## Additional Information

**How to cite this article**: Pasaje, C. F. *et al*. Selective inhibition of apicoplast tryptophanyl-tRNA synthetase causes delayed death in *Plasmodium falciparum*. *Sci. Rep.* **6**, 27531; doi: 10.1038/srep27531 (2016).

## Supplementary Material

Supplementary Information

## Figures and Tables

**Table 1:** Kinetics of *P. falciparum* TrpRSs.

protein	substrate	K_*m*_^app^ (μM)	V_*max*_^app^(nmole/min)
TrpRS^cyt^	ATP	622 ± 0.3	292 ± 0.01
	tryptophan	15.6 ± 0.6	77 ± 0.001
	tRNA		
	* **P. falciparum	0.020 ± 0.004	15 ± 0.01
	* E. coli*	1.2 ± 0.8	80 ± 0.1
	* S. cerevisiae*	0.23 ± 0.05	51 ± 0.02
TrpRS^api^	tryptophan	5.3 ± 1.2	29 ± 0.001
	tRNA		
	* **P. falciparum	0.04 ± 0.02	1 ± 0.001
	* E. coli*	0.12 ± 0.03	15 ± 0.001
	* S. cerevisiae*	3.6 ± 1.3	2 ± 0.001

^*^Total tRNA was used to determine kinetics of *Pf*  TrpRSs.

**Table 2:**
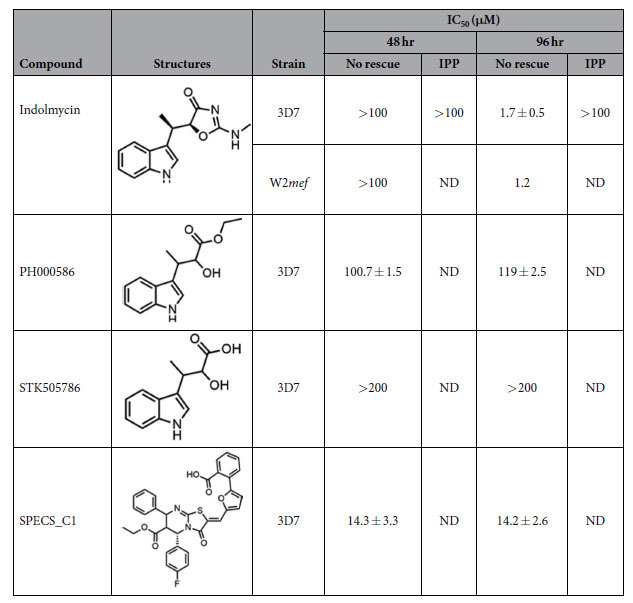
Antiplasmodial activity of bacterial-type TrpRS inhibitors against *P. falciparum*.

IC_50_ values were determined from corrected dose-response curves over time. ND, not determined.
