# Supplementary material for: Selective inhibition of apicoplast tryptophanyl-tRNA synthetase causes delayed death in Plasmodium falciparum
Source: Sci Rep. 2016 Jun 9;6:27531. doi: 10.1038/srep27531 (PMC4899734; doi:10.1038/srep27531)
Supplement: Supplementary Information [file srep27531-s1.doc]

Selective inhibition of apicoplast tryptophanyl-tRNA synthetase causes delayed death in *Plasmodium falciparum*

Charisse Flerida A. Pasaje^1^, Vanessa Cheung^1^, Kit Kennedy^1^, Erin E. Lim^1^, Jonathan B. Baell^2^, Michael D.W. Griffin^1^, Stuart A. Ralph^1*^

^1^Department of Biochemistry and Molecular Biology, Bio21 Molecular Science and Biotechnology Institute, The University of Melbourne, Victoria 3010, Australia

^2^Medicinal Chemistry, Monash Institute of Pharmaceutical Sciences, Monash University, 399 Royal Parade, Parkville, 3052 Victoria, Australia

*Correspondence and requests for materials should be addressed to S.A.R. (email:saralph@unimelb.edu.au)


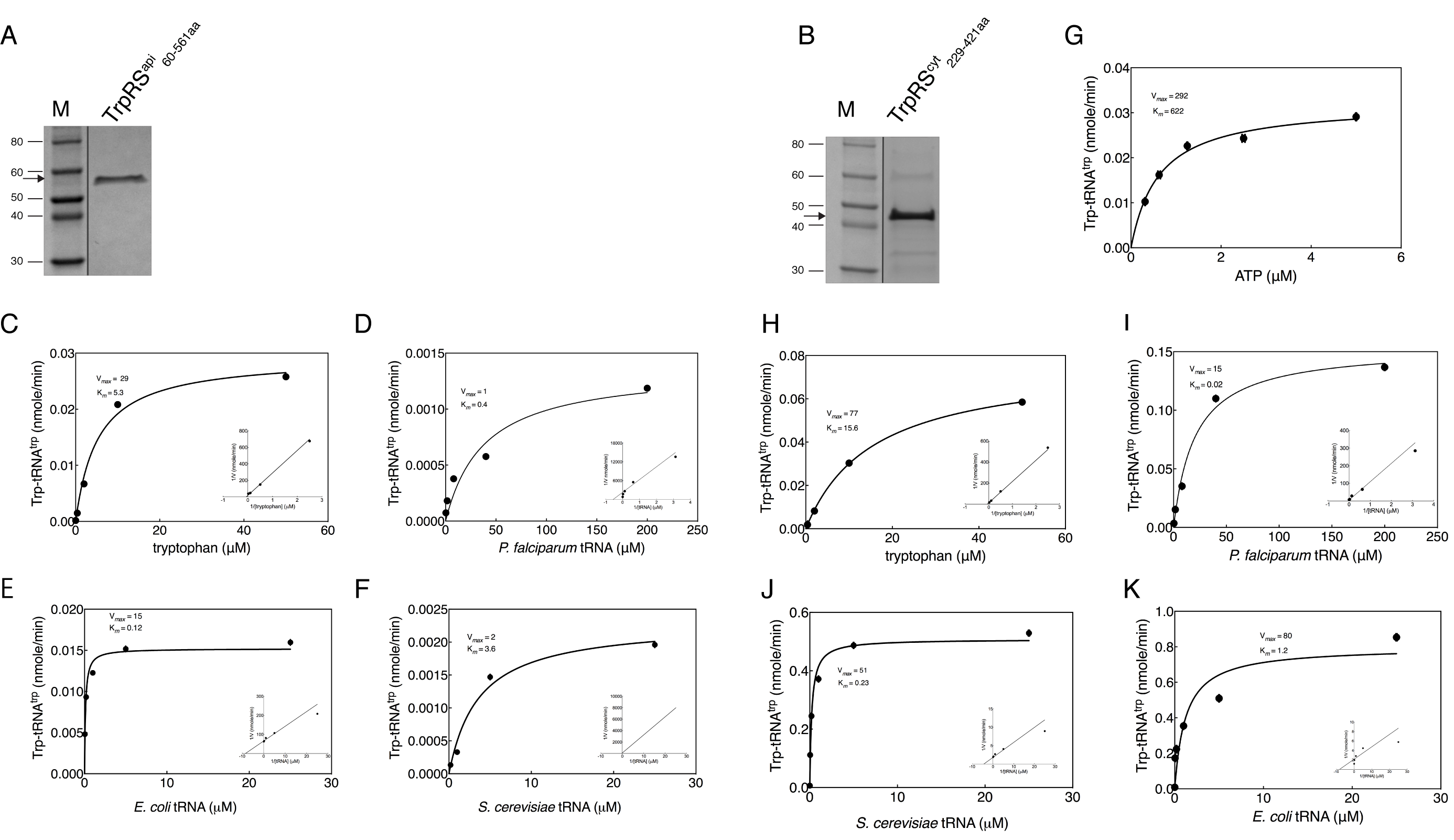
­

**Supplementary Figure S1. Steady state kinetics of TrpRS^api^.** Coomassie Brilliant Blue staining of **(a)** TrpRS^api^_pET21-21a(+) and **(b)** TrpRS^cyt^_AVA0421 expressed by auto-induction. Michaelis-Menten and Lineweaver-Burk plots of TrpRS^api^ aminoacylation at varying concentrations of ATP, tryptophan, and *P. falciparum, E. coli*, and *S. cerevisiae* tRNA as substrates of *Pf*TrpRS^api^ **(C-G)** and *Pf*TrpRS^cyt^ **(H-K).**

**
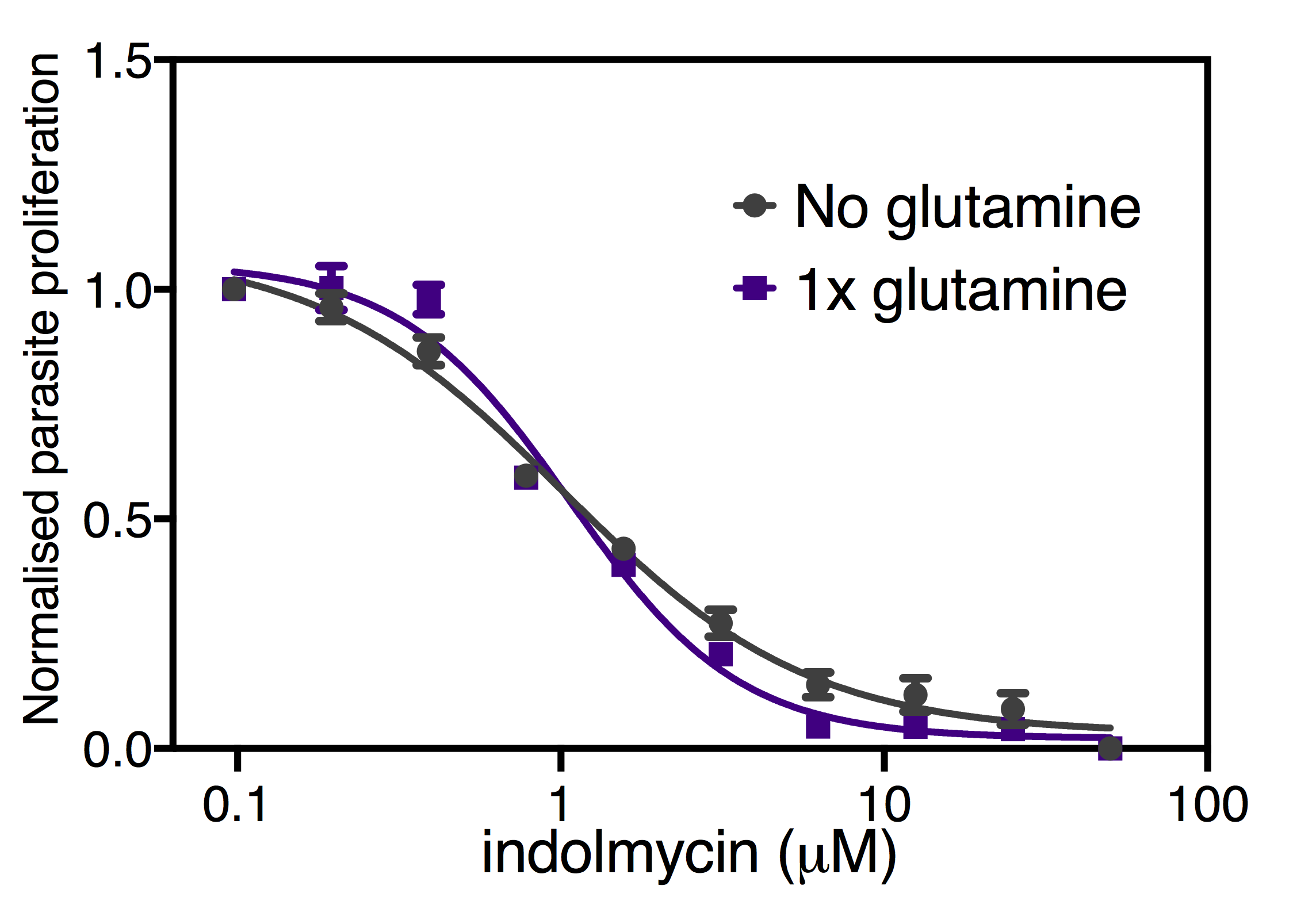
**

**Supplementary Figure S2.** Dose-response curve from SYBR-Green susceptibility assay determined 96 hrs after indolmycin treatment and at different concentrations of glutamine.

**Supplementary Table S1.** Summary of bacterial TrpRS inhibitors from the literature.

| Compound name | Target organism | Inhibitory effect (MIC_50_) | Reference |
| --- | --- | --- | --- |
| Indolmycin | *Helicobacter pylori* | ≤ 0.008-0.031 μg/mL | (Kanamaru et al., 2001) |
|  | *Escherichia coli* | 32 μg/mL | (Werner et al., 1976) |
|  | *Staphylococcus aureus* | 25 mg/mL | (Hurdle et al., 2004) |
|  |  |  |  |
| SPECS_compound 1 | *Staphylococcus epidermidis* | 6.25 μM | (Wu et al., 2007) |
|  | *Staphylococcus aureus* | 25 μM |

MIC_50_, Minimum Inhibitory Concentration required to inhibit the growth of 50% of organisms.
